# Biologic Activity and Biotechnological Development of Natural Products

**DOI:** 10.1155/2013/971745

**Published:** 2013-12-24

**Authors:** José Carlos Tavares Carvalho, Fabio Ferreira Perazzo, Leandro Machado, Didier Bereau

**Affiliations:** ^1^Campus Universitário Marco Zero do Equador, Rod Juscelino Kubitschek, KM-02, Jardim Marco Zero, 68.903-419 Macapá, AP, Brazil; ^2^Instituto de Ciências Ambientais, Químicas e Farmacêuticas, Universidade Federal de São Paulo, Rua Professor Artur Riedel, 275, Jd, Eldorado, 09.972-270 Diadema, SP, Brazil; ^3^Departamento de Tecnologia Farmacêutica, Faculdade de Farmácia-UFF, Rua Mário Viana, 523, Santa Rosa, 24.241-002 Niterói, RJ, Brazil; ^4^Université des Antilles et de la Guyane, Institut d'Enseignement Supérieur de la Guyane, Campus Universitaire de Troubiran, BP 792, 97337 Cayenne Cedex, France

Flora and Fauna diversity is an essential element of our environment. For millennia, human beings have used it for food as well as nonfood purposes such as medicine, dye-based tincture, or cosmetics, in direct application or after processing. Certain species, in view of their characteristics, have been domesticated, improved for outreaching the stadium of harvest or of hunting for family purposes, to be cultivated and fostered in large scale, entering market economy. They have thus become a source of wealth and economic development by establishing product branches and sophisticated exploitation.

However, it seems that, especially in tropical surroundings, this biodiversity is far from being valued adequately. Even though being fertile and abundant, little is known about it, in endemic and in cultivated wildlife species. Yet, knowing this biodiversity would allow exploiting it at its best while protecting it, which presents a major challenge.

In addition, currently there is a strong consumer demand; therefore there is a strong commercial pressure on

- improving the quality of consumer products,
- allocation of extracts, means purified substances, having quantified biological activities,
- a high level of safety for consumers.

Additionally, there are high expectations regarding environment-friendly exploitation of the natural resources, both for production and processing, as well as a fair return to the population having ceded their knowhow.

From 37 submissions, 24 papers are published in this special issue. Each paper was reviewed by at least two reviewers and revised according to reviews comment. The works presented in this special issue, related to different scientific fields, highlight the imperial need to characterize the natural substances physically and chemically and to evaluate their numerous biological activities.

Even if trace metals are important for living organisms in order to stabilize protein structures, to facilitate electron transfer reactions, and to catalyse enzymatic reactions, they can also lead to toxicity, mainly for oxidative stress reason. Fungi may be the alternative solution to resins because of their ability to tolerate and detoxify metals. In N. M. Abdel-Monem et al.'s paper *“Pretreatment hepatoprotective effect of the marine fungus derived from sponge on hepatic toxicity induced by heavy metals in rats,” *the authors prove a pretreatment hepatoprotective effect of the extract of marine-derived fungus *Trichurus spiralis* Hasselbr. isolated from *Hippospongia communis* sponge on the hepatotoxicity, following heavy-metal mixture's administration (Cd, Co, Hg, Ni chloride, and Pb acetate 0.25 mg) investigated in rats for a period of 7 days. They demonstrated that marine-derived fungus extract (T.S) possesses a hepatoprotective property due to its proven antioxidant and free radical scavenging properties.

Nowadays, reduction in release of methane into the environment is required for all countries. Lovastatin, which is a natural polyketide synthesized by *Aspergillus terreus* and *Pleurotus ostreatus* (oyster mushroom), may play this important role in rumens by inhibiting HMG-CoA reductase activity, a key enzyme involved in the formation of isoprenoid building blocks that are essential for cell membrane synthesis in methanogenic Archaea. Because of its expensive cost for large scale use in agriculture, an alternative could be the study done in M. F. Jahromi et al.'s paper *“Lovastatin in Aspergillus terreus: fermented rice straw extracts interferes with methane production and gene expression in Methanobrevibacter smithii.” *The authors described a series of experiments designed to test the hypothesis that unpurified lovastatin secreted by *Aspergillus terreus* in fermented rice straw extracts (FRSE) inhibits growth and CH4 production in a representative methanogen—*Methanobrevibacter smithii* (DSM 861). FRSE stronger effect when compared to commercial lovastatin was demonstrated. Thus, it is feasible to use *A. terreus*-fermented rice straw (an industrial byproduct) as feed additive in ruminants.

Ferulic acid (4-hydroxy-3-methoxycinnamic acid) (FA) is a ubiquitous phenolic acid in the plant kingdom. It plays an important role, like other flavonoids, in health owing to its potential biological activities. However, studies revealing its adverse effects are increasing. In C. C. Peng et al.'s paper *“Cytotoxicity of ferulic acid on T24 cell line differentiated by different microenvironments*,*”* the authors tried to understand the biological behaviors and cytotoxicity involved in 3D-cells culture (native three-dimensional structure) compared to 2D-monolayers when FA was occurring. FA (2 mM) in the 3D-culture revealed significantly higher cytotoxicity than the 2D-culture suggesting that biological significance may be implicated by using either 3D- or 2D-culture model.

Inflammatory which is responsible for morbidity and mortality in the world can be treated either with nonsteroidal anti-inflammatory drugs (NSAIDs) or steroidal drugs. Both have proven to be effective but can have negative side effects. In a way to find new molecules with anti-inflammatory activity but with fewer side effects, in C. P. González et al.'s paper *“Anti-inflammatory activity and composition of Senecio salignus Kunth,”* the authors investigated the anti-inflammatory activity of *Seneco salignus*, a plant issued from traditional medicine, by induced edemas and the composition of its active fraction by GC-MS. An active fraction was obtained from chloroform extract in both anti-inflammatory tests in which a total of 185 compounds were isolated.

An endemic plant of the Brazilian Atlantic Forest and widely spread in the sandbanks of “Restinga de Jurubatiba” National Park, *Neomitranthes obscura* (DC.), was studied in order to evaluate chemical composition of essential oils obtained from specimens with different fruit color by GC and GC/MS analysis. In R. R. Amaral et al.'s paper *“Essential oils from fruits with different colors and leaves of Neomitranthes obscura (DC.) N. Silveira: an endemic species from Brazilian Atlantic Forest,”* the authors demonstrated the predominance of sesquiterpenes (in leaves and yellow fruits essential oils), meanwhile monoterpenes were found in black fruits oils. All these results were suggesting the occurrence of unless two different varieties for this species.

In hypertension and cardiovascular diseases which affect developed countries, Angiotensin I-converting enzyme (ACE) plays important physiological role in blood pressure regulation. The consumption of ACE inhibitory peptides may be useful. In J. C. Wu et al.'s paper *“Preparation of ACE inhibitory peptides from Mytilus coruscus hydrolysate using uniform design,” * the authors investigated the ACE inhibitory potential of peptides isolated from mussel, *Mytilus coruscus*, according to variable factors such as protease concentration, hydrolysis time, PH, and temperature. Uniform Design, a new statistical experimental method, was operated. A high percentage of lysine, leucine, glycine, aspartic acid, and glutamic acids were found suggesting nutraceuticals and pharmaceuticals purposes.

Rubiaceae family is well known for the presence of secondary metabolites with pharmacological potential among them antibacterial properties. In D. Martins et al.'s paper *“Triterpenes and the antimycobacterial activity of Duroia macrophylla Huber (Rubiaceae),”* an occurring species in the Amazon Forest, *Duroia macrophylla*, was studied in order to evaluate the antimycobacterial activity of its extracts and isolate and identify the substances present. The authors found major biological activity against *Mycobacterium tuberculosis* in leaves dichloromethane extract. Molecules belonging to terpenes, alkaloids, and flavonoids were also isolated; oleanic and ursolic acids were reported for the first time in *Duroia* genus.

Flavonoids, and especially anthocyanins, are well known for their biological activities: antiproliferative, hypoglycemic, antioxidant, and antiobesity effects. Anti-inflammatory properties are also well described in several biochemical pathways. In N. M. A. Hassimotto et al.'s paper *“Inhibition of carrageenan-induced acute inflammation in mice by oral administration of anthocyanin mixture from wild mulberry and cyanidin-3-glucoside,”* attention has been paid to an anthocyanin-enriched (AG) fraction from mulberry and cyanidin-3-glucoside (C3G) to evaluate their potential against two acute inflammations induced by carrageenan in mice (peritonitis and paw oedemas). Positive results have been demonstrated suggering the use of AG and C3G for prophylactic or therapeutic purposes.

Interest in natural products and especially in bee products as honey, royal jelly, pollen, and propolis is increasing. This latter showed various biological properties, among them, immunomodulatory, antibacterial, fungicidal, anti-inflammatory, healing, anesthetic, and anticarcinogenic effects. In M. M. Possamai et al.'s paper *“Brazilian propolis: a natural product that improved the fungicidal activity by blood phagocytes,” * the authors focused on the propolis of a Brazilian stingless bee (*Meliponae* subfamily) in order to evaluate the potential effects of its adsorbed form on polyethylene glycol (PEG) on the activity of human phagocytes against *Candida albicans*. Immunostimulatory effects were demonstrated suggesting this adsorbed form of propolis for many other therapeutic uses.

*Ampelozizyphus amazonicus* Ducke (belonging to Rhamnaceae family) is a popular plant from Amazonian folks and is described by ethnobotanists as useful in the treatment and prevention of malaria. As no direct action on *Plasmodium* blood stage forms was demonstrated, in L. M. T. Peçanha et al.'s paper *“Immunobiologic and antiinflammatory properties of a bark extract from Ampelozizyphus amazonicus Ducke,”* the authors paid attention to the immunological response of *Plasmodium chabaudi*-infected mice, treated by an aqueous extract of this plant. Immunomodulatory and anti-inflammatory properties were found, maybe due to the presence of a complex dammarane-type saponin.

Mushrooms are well implanted in Asian, therapies, specifically for their medicinal uses. *Sparassis crispa*, known as cauliflower mushroom, is getting more and more popular thanks to not only its taste but also its therapeutic applications. In T. Kimura's paper *“Natural products and biological activity of the pharmacologically active cauliflower mushroom Sparassis crispa,”* the author presents an overview of the pharmacological properties of this mushroom and the mechanisms of action of its bioactive components. Specifically, the immunomodulatory mechanisms of beta-glucan, an important present molecule, are well described.

In response to an increasing interest of consumers for functional foods, studies using probiotic organisms are more frequent. In J. E. Park et al.'s paper *“Lactobacillus plantarum Lg42 isolated from Gajami Sik-Hae inhibits adipogenesis in 3T-L1 adipocyte,” * the authors investigated a popular Korean fermented fish product (Gajami Sik-Hae), paying special attention to the beneficial influence of the herein *Lactobacillus plantarum* lactic acid bacteria (GLAB) on lipid accumulation in adipocytes by regulating the expression of adipogenesis-related genes in differentiated 3 T3-L1 cells. Inhibitory effects of the GLAB by modulating the expression of adipogenic transcription factors were demonstrated, suggesting the antiobesity property of this fermented seafood.

Duchenne muscular dystrophy (DMD) is an X-linked genetic disorder resulting in a defect in the muscle membrane protein called dystrophin. Its consequences may be a loss of ability to walk and cardiac function and respiratory muscles strength reductions. In D. Feder et al.'s paper *“Hormonal receptors in skeletal muscles of dystrophic Mdx mice,”* the authors, for the first time, investigated the gene expression of hormone receptors in different muscles (dystrophic and healthy) in mdx and C57BL6 mice. Dystrophic muscles have some significant differences in hormone receptor expression when compared to normal mice suggesting more studies to be enhanced on this path in a way to understand the events related to this pathophysiology.

Atherosclerosis is a cardiovascular disease resulting from the rupture of an atherosclerotic plaque. Its stabilization and the inhibition of its progression with macrophages apoptosis are of paramount importance. In F. Wang et al.'s paper *“The sonodynamic effect of curcumin on THP-1 cell-derived macrophages,”* the authors chose a new method with ultrasound using curcumin as a sonosensitizer on THP-1 derived macrophages: the sonodynamic therapy (SDT). They concluded that curcumine by SDT decreases macrophages viability and induces their apoptosis or necrosis. Both loss of mitochondrial membrane potential and morphological changes of cytoskeleton were apparent. So, curcumin could be a new sonosensitizer in a promising treatment for atherosclerosis.

Tandem repeats of proteins and peptides are known to have many stabilizing functions. Thymosin alpha 1 (T*α*1), which is composed of 28 amino acids, has immune-modulatory and antitumor properties. In X. C. Xue et al.'s paper *“Construction, expression, and characterization of thymosin alpha 1 tandem repeats in Escherichia coli,”* the authors created, in *E. coli* TOP10, the molecule T*α*1*③*, which is composed of three repeated copies of T*α*1 and is fuse-expressed with thioredoxin. Its biological activity on T lymphocyte proliferation and IL-2R expression was significant. This work is an efficient tool for a large scale production of this active protein.

2, 3, 7, 8-Tetrachlorodibenzo-p-dioxin (TCDD) is a known environmental contaminant involving the formation of CYP1A1, a carcinogen-activating enzyme. Harmine and harmaline, *β*-carboline alkaloids, were already described in the literature as molecules with pharmacological activities. In a previous work, M. A. M. El Gendy et al. had already demonstrated the inhibitory effects of these alkaloids on CYP1A1 in vitro. In the paper *“Harmine and harmaline downregulate TCDD-induced Cyp1a1 in the livers and lungs of C57bl/6 mice” *by M. A. M. El Gendy and A. O. S. El-Kadi, this work is enhanced in in vivo conditions on mice livers and lungs. Promising results were found so that harmine and harmaline could be purposed as good candidates in the strategy against carcinogenesis.

Female reproductive ability can be affected by several parameters: production and transport of gametes, endocrine system, sexual behavior, gestation, parturition, lactation, and alterations in other functions. *Pradosia huberi*, a well known Sapotaceae plant from the Brazilian folk medicine, can present potential toxic effects next to its anti-inflammatory properties. In A. O. B. Rocha et al.'s paper *“Evaluation of the toxicity of Pradosia huberi extract during the preimplantation in Wistar rats,”* the authors paid attention to the effects of hydroalcoholic extracts of *P. huberi* during the uterine implantation time of Wistar rats. Compromising results were found on the reproductive ability during the embryonic preimplantation phase, suggesting a possible toxic effect upon the reproductive system of Wistar rats.

*Manilkara subsericea* is a widely spread species on the sandbanks of eastern Brazil with food and construction local uses. Potential biological activities and chemical compositions (including triterpenes, saponins, and flavonoids) were already reported in the literature for the genus *Manilkara*. In C. P. Fernandes et al.'s paper *“Triterpene esters and biological activities from edible fruits of Manilkara subsericea (Mart.) Dubard, Sapotaceae,”* the authors focused on antibacterial and cytotoxicity of extracts from *Manilkara subsericea* and chemically characterized the hexanic extract from its edible fruits. Beta- and alpha-amyrin caproates and caprylates were reported for the first time for this species. Antimicrobial activity against *Staphylococcus aureus* was relevant. And weak toxicity against Vero cells was also demonstrated.

Given the lack of knowledge concerning the nutrient contents of fish and shellfish species in Malaysia, in N. Abd Aziz et al.'s paper *“Quantitative determination of fatty acids in marine fish and shellfish from warm water of Straits of Malacca for nutraceutical purposes,”* the authors investigated through qualitative and quantitative nutritional analysis 20 species issued from Straits of Malacca. Fatty acids composition and amount of each fatty acid were determined. Most samples contained fairly high amounts of polyunsaturated fatty acids (PUFAs), meanwhile some species showed significantly high amounts of eicosapentaenoic acid (EPA), docosahexaenoic acid (DHA), alpha-linolenic acid (ALA), and omega-3 fatty acids. The polyunsaturated-fatty-acids/saturated-fatty-acids (P/S) ratios for most samples were high. These promising results could be useful for nutraceutical purposes.

*Allium hirtifolium* Boiss, known as Persian shallot, is an Iranian native spice with traditional uses, specifically in food uses. The numerous biological activities attributed to both famous plants issued from the same genus (garlic and onion) were relevant enough so studies be investigated on this species. In S. Ismail et al.'s paper *“Chemical composition and antibacterial and cytotoxic activities of Allium hirtifolium Boiss,”* the authors paid attention to the chemical composition, antibacterial and cytotoxic effects of the spice. Hydromethanolic extract, through GC/MS analysis, revealed the main presence of 9-hexadecenoic acid, 11,14-eicosadienoic acid, and n-hexadecanoic acid. And persian shallot, because of its efficiency against 10 different species of pathogenic bacteria, could be considered as a safe and strong antibacterial agent.

Because of the increasing bacteria antibiotic resistance, plants could play an alternative role. *Amburana cearensis* A. C. Smith and *Anadenanthera macrocarpa* (Benth.) Brenan are well known from folks of South America. In F. G. Figueredo et al.'s paper *“Modulation of the antibiotic activity by extracts from Amburana cearensis A. C. Smith and Anadenanthera macrocarpa (Benth.) Brenan,”* the authors investigated the phytochemical composition and the antibacterial and modifying antibiotic activities of these plants. Their ethanolic extracts demonstrated antibacterial action due to the presence of several antibacteria responsible for modulatory effects. The use of these natural products combined with aminoglycosides in order to increase their antimicrobial potential against multiresistant microorganisms could be a serious alternative.

*Pothomorphe umbellata *L. (*Piperaceae*) is well know in Brazil for its different pharmacological activities (anti-inflammatory, analgesic, antiulcer, gastroprotective, antimalarial, and antioxidant) mainly due to a phenolic compound located in the vegetable roots and leaves: the 4-nerolidylcathecol. In A. P. Lopes et al.'s paper *“Antioxidant and cytotoxic effects of crude extract, fractions and 4-nerolidylcathecol from aerial parts of Pothomorphe umbellata L. (Piperaceae),”* the authors paid attention to the crude ethanolic and aqueous-ethanolic extracts, sterol fraction, and 4-nerolidylcathecol of the plant in order to evaluate its antioxidant activity and its cytotoxic effect in HL-60 cells. Significant antioxidant potential and low toxicity were found to be corroborating the safe and effective use of *P. umbellata* by folklore medicine.

Breast cancer causes the highest percentage of the cancer deaths in women worldwide (in both developing and developed countries). Next to the existing options of treatment (mostly correlated to serious side effects), bioactive components of algae can be a potential alternative. In S. E. Nigjeh et al.'s paper *“Cytotoxic effect of ethanol extract of microalga, Chaetoceros calcitrans, and its mechanisms in inducing apoptosis in human breast cancer cell line,”* the authors investigated the cytotoxic effect and apoptosis mechanism of crude ethanol extracts of an indigenous microalga from Malaysia, *Chaetoceros calcitrans,* on human breast cell lines. Exposure of MCF-7 and MCF-10A cells to crude ethanol extracts of *C. calcitrans* (EEC) resulted in cell number decrease through induction of apoptosis or modulation of gene expression related to cell cycle. *Chaetoceros calcitrans* could be a chemopreventive agent for breast cancer treatment.

Diabetes is a worldwide epidemic which is characterized by a disturbance of carbohydrate, fat, and protein metabolism resulting from defects in insulin secretion, insulin action, or both. Treatment may be operated by several oral antihyperglycemic agents, but side effects occur. In D. Cheng et al.'s paper *“Antihyperglycemic effect of Ginkgo biloba extract in streptozotocin-induced diabetes in rats,”* the authors evaluated the antihyperglycemic effects of a *Ginkgo biloba* extract (GBE), a beneficial plant in Chinese medicine, on streptozotocin (STZ) induced diabetes in rats. Increase in body weight and antioxidant ability and decrease in blood glucose, lipid profile, and lipid peroxidation were noticed, suggesting GBE as supplement or adjunct treatment for diabetics.

These papers are real state of the art which emphasize potential uses for natural products. Originated from temperate or tropical climate, natural substances are applied in various domains like food, pharmaceutics, or cosmetics purposes. They are key elements for the future development of the countries they originate from. We hope that this special issue would attract a major attention of the peers.

